# Cell adhesion-related gene somatic mutations are enriched in aggressive papillary thyroid microcarcinomas

**DOI:** 10.1186/s12967-018-1642-0

**Published:** 2018-10-01

**Authors:** Jianlu Song, Shouxin Wu, Xiaotian Xia, Yu Wang, Youben Fan, Zhili Yang

**Affiliations:** 1 Department of General Surgery, Shanghai Jiao Tong University Affiliated Sixth People’s Hospital, 600 Yi-Shan Road, Shanghai, 200233 People’s Republic of China; 2 Zhangjiang Center for Translational Medicine, Shanghai Biotecan Medical Diagnostics Co., Ltd, Shanghai, 201203 People’s Republic of China

**Keywords:** Aggressiveness, Cell adhesion, Whole-exome sequencing, Somatic mutation, Papillary thyroid microcarcinoma

## Abstract

**Background:**

Approximately half of the documented increases in differentiated thyroid carcinoma is due to identification of papillary thyroid microcarcinomas (PTMCs). Knowing whether PTMC is aggressive is required for proper treatment, but until now, there has been no method for assessing these traits and understanding the underlying mechanisms for aggressiveness.

**Methods:**

We performed whole-exome sequencing of 16 PTMCs and matched normal thyroid tissues and GO/KEGG analysis to study genetic alterations and biological consequences associated with aggressive PTMCs, and then sequenced these genes using a next-generation gene-panel approach in an additional 70 PTMC samples including aggressive (n = 50) and non-aggressive (n = 20) groups.

**Results:**

We identified 254 somatic mutations of 234 genes, for which 178 mutations in 168 genes were found in the aggressive group, and 76 mutations in 74 genes were found in the non-aggressive group. Several recurrent mutations in *BRAF*, *VCAN*, *ALDH1L1*, and *MUC5B* were identified, and many novel but infrequent mutations in other genes were also found. The aggressive cohort had more mutational burdens than the non-aggressive group (*P *= 0.004). Nonsynonymous mutations of 13 genes (*MUC5B*, *TNN*, *SSPO*, *PPFIA1*, *PCDHGA2*, *ITGA8*, *ITGA4*, *DCHS1*, *CRNN*, *ROCK1*, *RELN*, *LAMC2*, and *AEBP1*) were involved in cell adhesion, and these were only present in the aggressive group. Targeted sequencing of these genes revealed significant enrichment in the aggressive group (*P *= 0.000004).

**Conclusion:**

PTC may have evolved from PTMC due to sharing similar gene mutations, and the accumulation of such mutations promoted the aggressiveness of PTMC. Gene mutants associated with cell adhesion may be used to predict PTMC aggressiveness and allow more selective treatment.

## Background

The incidence of differentiated thyroid carcinoma (DTC) is increasing rapidly in many countries, but its mortality remains low and stable [1, 2]. Approximately 50% of the increase in DTC is due to the identification of papillary thyroid microcarcinoma (PTMC), which is a thyroid papillary carcinoma (PTC) tumor ≤ 1 cm in diameter. Although most PTMC have an indolent clinical course, some are high-risk for aggressiveness and tumor invasion, metastasis, and patient mortality [3, 4]. Certain patient traits, such as being male, older than 45 years-of-age, having tumors > 5 mm that are multifocal with extrathyroidal extensions and lymphovascular invasion, are thought to be aggressive markers for PTMC [5, 6]. However, despite combining multiple clinical factors, the specificity and positive predictive value of these models are not sufficient for providing useful preoperative risk stratification [7]. Furthermore, many potentially important risk factors can only be determined postoperatively based on thyroid pathology [8]. Thus, molecular signatures may be used at presentation to predict whether a PTMC will be aggressive.

It was reported that the BRAF^V600E^ mutation is frequent in PTMC (30–67%) [9]. Recent studies suggest that BRAF^V600E^-mutation-positive PTMC are more likely to be aggressive and have extrathyroidal tumor extensions, lymph node metastases, and recurrence [10], but studies suggest little use for *BRAF* mutation testing to stratify PTMC tumor risk as there is no correlation between *BRAF*-positive primary PTMCs and more aggressive or recurrent disease [11]. The discovery of *TERT* promoter mutations in thyroid cancer [12] were found to be useful, as 9.4% of PTC have *TERT* promoter mutations that are associated with older patients, higher MACIS scores and high risk of recurrence [13]. For PTMC, *TERT* promoter mutations are uncommon (4.7%) and not correlated with unfavorable clinical features [14].

Major genetic alterations in PTC have been evaluated with whole-exome sequencing (WES) by the Cancer Genome Atlas (TCGA) Research Network [13] and others [15]. Lately, several comprehensive and integrated analyses of thyroid cancer were reported across the Cancer Genome Atlas [16, 17]. However, to our knowledge, PTMC was not a focus of these studies. Thus, we require a better predictor for aggressive PTMC, and next-generation sequencing may accelerate clinical research in this regard by enabling analysis of genomes and identification of a gene mutation signature in aggressive PTMC.

We used WES for 16 matched PTMCs to investigate genetic alterations and biological consequences and targeted sequencing to verify these genes in additional aggressive PTMC samples. PTC may have evolved from PTMC that shared similar gene mutations. Aggressive PTMC had more mutational burdens and were enriched for specific sets of mutational genes related to cell adhesion, and this may be used to treat PTMC.

## Methods

### Clinical samples

Tumors and matched normal thyroid tissues for sequencing were collected during surgical resection from 86 patients diagnosed with PTMC at Shanghai Jiao Tong University Affiliated Sixth People’s Hospital. Unilateral central neck dissection was performed in all cases, regardless of clinical lymph node involvement, and selective lateral lymph node dissection was performed for patients with clinical lateral lymph node metastases. Histopathology of tumors and related lymph node samples were confirmed pathologically. Patients were divided into aggressive and non-aggressive groups according to whether the primary lesion had extrathyroidal extensions and lymph node metastasis. Cohort data of WES in 16 paired samples appear in Table 1, and 70 other samples were used for target sequencing. No individual from whom fresh cancer tissues were collected received preoperative chemotherapy or radiotherapy. This study was approved by the Ethics Committee of Shanghai Jiao Tong University Affiliated Sixth People’s Hospital. Informed consent was obtained.

**Table 1:** Clinical and pathological characteristics of PTMC tumor samples subjected to WES

Case	Age	Gender	Tumor size (cm)	Multifocality	Extrathyroidal extension	Lymph node metastasis	Distance of metastasis	Histological subtypes
T1	37	F	0.6	1	No	No	No	C-PTC
T2	72	F	0.3	1	No	No	No	C-PTC
T3	43	F	0.7	1	No	No	No	C-PTC
T4	41	F	0.6/0.5/0.5	3	No	No	No	C-PTC
T5	28	F	0.5	1	No	No	No	C-PTC
T6	45	F	0.7	1	No	No	No	C-PTC
T7	37	F	0.5	1	No	No	No	C-PTC
T8	67	M	0.4/0.2	2	No	No	No	C-PTC
T9	34	F	0.6	1	Yes	No	No	FV-PTC
T10	26	F	0.5	1	Yes	No	No	C-PTC
T11	37	F	0.6/0.3/0.2	3	No	Yes	No	C-PTC
T12	45	F	0.4	1	No	Yes	No	C-PTC
T13	31	F	0.2	1	No	Yes	No	C-PTC
T14	39	F	0.6	1	No	Yes	No	C-PTC
T15	28	F	0.5	1	No	Yes	No	C-PTC
T16	56	M	0.8	1	No	Yes	No	C-PTC

*C-PTC* classical papillary thyroid carcinoma, *FV-PTC* follicular variant papillary thyroid carcinoma

### DNA extraction and quality control

GDNA from fresh frozen tissues was extracted by GeneRead DNA Kit (Qiagen, Hilden, Germany). Quantity and purity of gDNA were assessed by Qubit 3.0 Fluorometer (Invitrogen, Carlsbad, CA) and NanoDrop ND-1000 (Thermo-Scientific, Wilmington, DE). Fragmentation status were evaluated by the Agilent 2200 TapeStation system using the Genomic DNA ScreenTape assay (Agilent Technologies, Santa Clara, CA) to produce a DNA Integrity Number (DIN). An additional quality control (QC) step to assess fresh frozen tissue DNA integrity was performed using a multiplex PCR approach. Briefly, 30 ng of gDNA were amplified using three different- primers for the GAPDH gene (200–400 base pair), and PCR products was measured using an Agilent 2100 Bioanalyzer (Agilent Technologies, Santa Clara, CA). Then, to estimate fresh frozen tissues gDNA fragmentation, we evaluated average yield ratio (AYR) values, calculated by yield ratio of each amplicon compared with a reference DNA (Promega Madison, WI).

### WES library preparation and hybridization capture

A total of 30 ng of each gDNA sample based on Qubit quantification were mechanically fragmented on an E220 focused ultrasonicator Covaris (Covaris, Woburn, MA). Sheared gDNA (200 ng) was used to perform end repair, and A-tailing and adapter ligation with either Agilent SureSelect XT (Agilent Technologies, Santa Clara, CA) or KAPA library preparation kits (Kapa Biosystems Inc., Wilmington, MA) was performed according to the manufacturer’s instructions. Subsequently, libraries were captured using Agilent SureSelect Human All Exon v.6 (Agilent Technologies, Santa Clara, CA) probes and amplified.

### Illumina sequencing

After QC and quantification by Agilent 2100 Bioanalyzer (Agilent Technologies, Santa Clara, CA) and Qubit 2.0 Fluorometer (Invitrogen, Carlsbad, CA), libraries were sequenced on an Illumina HiSeq 2500 platform (Illumina Inc, San Diego, CA) High output mode using 2 × 150 cycles was performed with TruSeq SBS v3 chemistry. For each library preparation type, 10 samples were loaded in a single lane of a flow-cell v3.

### Next-generation gene-panel sequencing

Seventy DNA samples were analyzed for target-capture sequencing of 13 genes with the SureSelect Target Enrichment Kit on an Illumina HiSeq 2500 platform. The average coverage of the targeted region was 600×, and 95% of the target was covered at > 50×. Sequencing reads were aligned to the human genome (NCBI build 37) with the BWA algorithm on default settings. Finally, 70 cases passed internal quality control and quality matrix and were included in further analyses.

### Bioinformatic analysis

BCL files generated by Illumina for WES samples were converted to FASTQ format by CASAVA software (v.1.8.1, Illumina) and aligned to the human reference genome hg19 with the Burrows-Wheeler Aligner. Local realignment, PCR duplicate marking, base-quality recalibration, and calculation of coverage metrics were performed with GATK and Picard tools. Somatic SNVs and indels were called with MuTect2. Identified variants were annotated with ANNOVAR. We considered variants that passed the standard MuTect2 filters, and excluded common SNPs with a minor allele frequency of > 0.01 as recorded in the NHLBI exome sequencing project, 1000 Genomes and Exome Aggregation consortium. We also excluded variants in non-coding regions and synonymous variants. We used DAVID for GO and KEGG pathway enrichment analysis.

### Statistical analysis

Statistical analysis was performed using IBM SPSS Statistics 22.0 (SPSS, Chicago, IL). Demographic and clinicopathologic characteristics, as well as mutation burden and frequency, were compared using Fisher’s exact test for categorical variables, and a Student’s *t* test was used for continuous variables. A two-tailed P < 0.05 was considered statistically significant.

## Results

### Demographic and clinicopathologic characteristics of samples

In the WES cohort (Table 1), median age was 36 (range 26–56) and 42 (range 28–67) years for aggressive and non-aggressive PTMCs, respectively (*P *= 0.17). Female/male ratios were 7/1 (aggressive group) and 7/1 (non-aggressive group) (*P *> 0.05). In the validating cohort (Table 2), there was no significant difference in demographic and clinicopathologic characteristics between groups (P > 0.05). All patients were of Han nationality, and there was no statistical bias.

**Table 2:** Demographic and clinicopathologic characteristics of 70 PTMCs in validation cohort

Parameters	Non-aggressive group (n = 20)	Aggressive group (n = 50)	*P* value
Age (years, median)	38 (25–56)	39 (24–60)	0.71
Female/male ratio	16/4	38/12	0.49
Size (cm)	0.5 ± 0.1	0.6 ± 0.1	0.44
Histological type (C-PTC/FV-PTC)	19/1	47/3	0.67

*C-PTC* classical papillary thyroid carcinoma, *FV-PTC* follicular variant papillary thyroid carcinoma

### Identification of somatic mutations in PTMCs

**Fig. 1:**
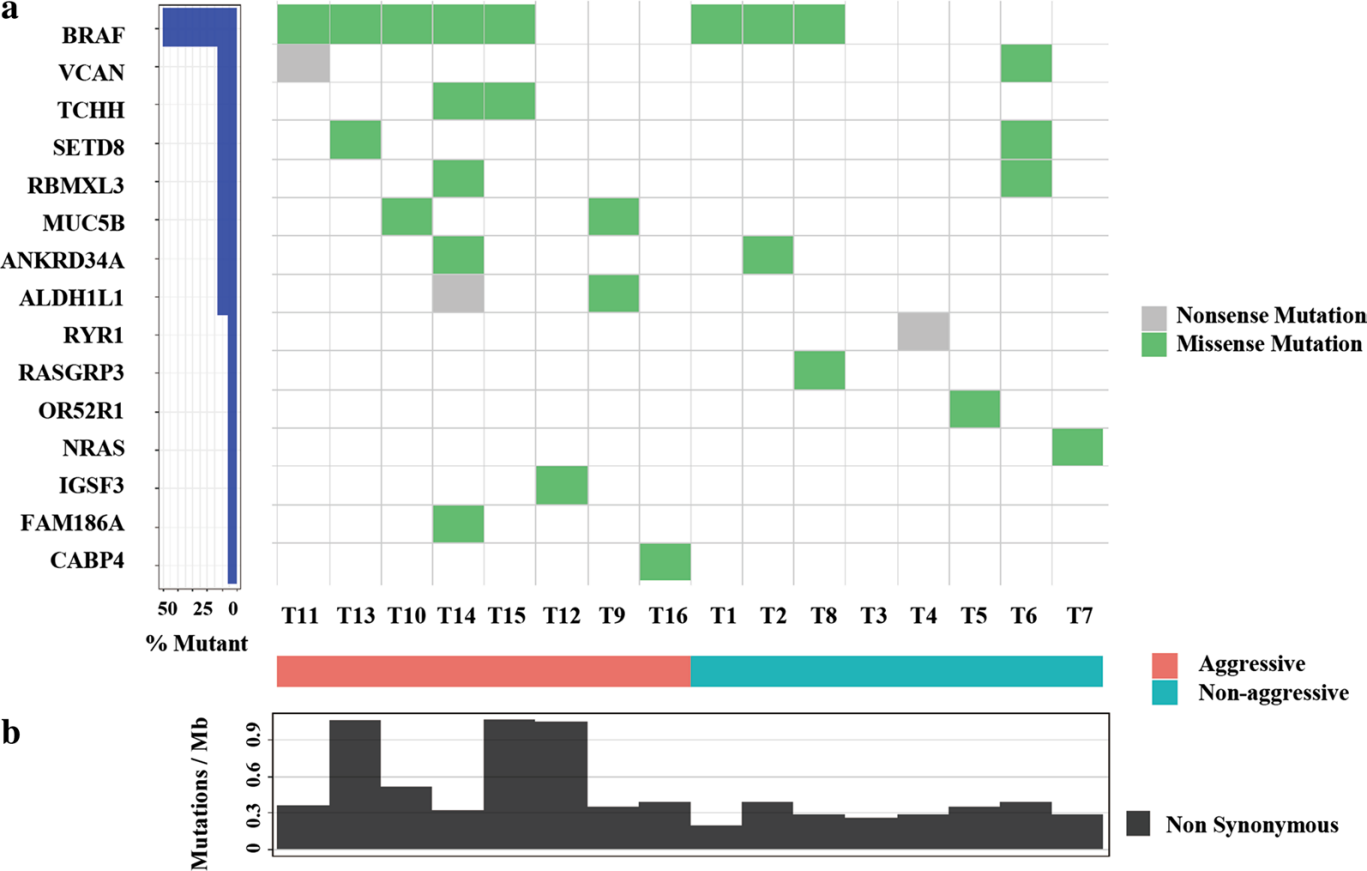
Mutational landscape and tumor mutation burden in WES cohort. **a** Frequency and types of mutations in 15 genes identified by WES. Different mutations and subtypes are colored. Mutations are shown on left (%). **b** Tumor mutation burden (mutations/MB) in 16 PTMC

We characterized 16 cases with WES, half of which were invasive and metastatic (aggressive) and 8 which were not (non-aggressive). Figure 1a shows data from this analysis. We also found novel mutations in different genes occurring at low frequency. Figure 1a shows these data. For 16 primary tumors, their somatic mutation burden (mutations/MB) data appear in Fig. 1b. PTMCs in our cohort of 16 cases showed 0.49 (range 0.19–1.34) median nonsynonymous somatic mutations per Mb, 50% mutation frequency of BRAF^V600E^, and 6.2% of mutation frequency of NRAS, which are comparable to what were reported in PTCs by the Cancer Genome Atlas Research Network (0.41 somatic mutations per Mb, 59.7% BRAF^V600E^, and 8.5% NRAS).

### Frequently mutated pathways and biological consequences in PTMC

**Fig. 2:**
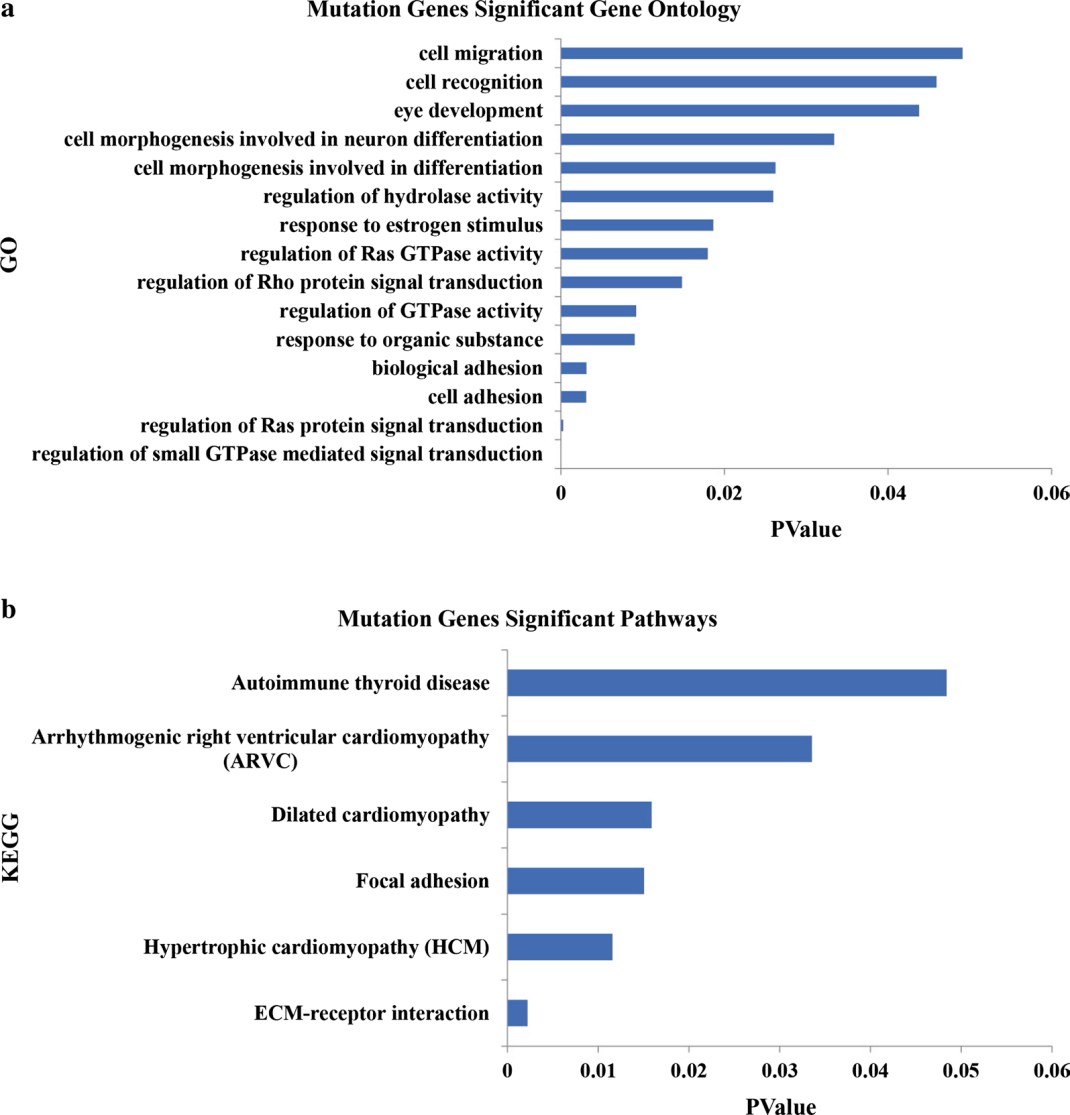
Key pathways and biological functions analysis of a 16 PTMC cohort. **a** Key pathways analysis of 16 PTMC. **b** KEGG analysis of mutations in 16 PTMC

Pathway analysis data appear in Fig. 2a, and most pathways involved were cancer-related (Fig. 2b). Thus, most major gene mutations identified here would contribute to the tumorigenesis of PTMC by regulating important cancer biological functions and signaling.

### Analysis of the mutation burden of the aggressive PTMC

**Fig. 3:**
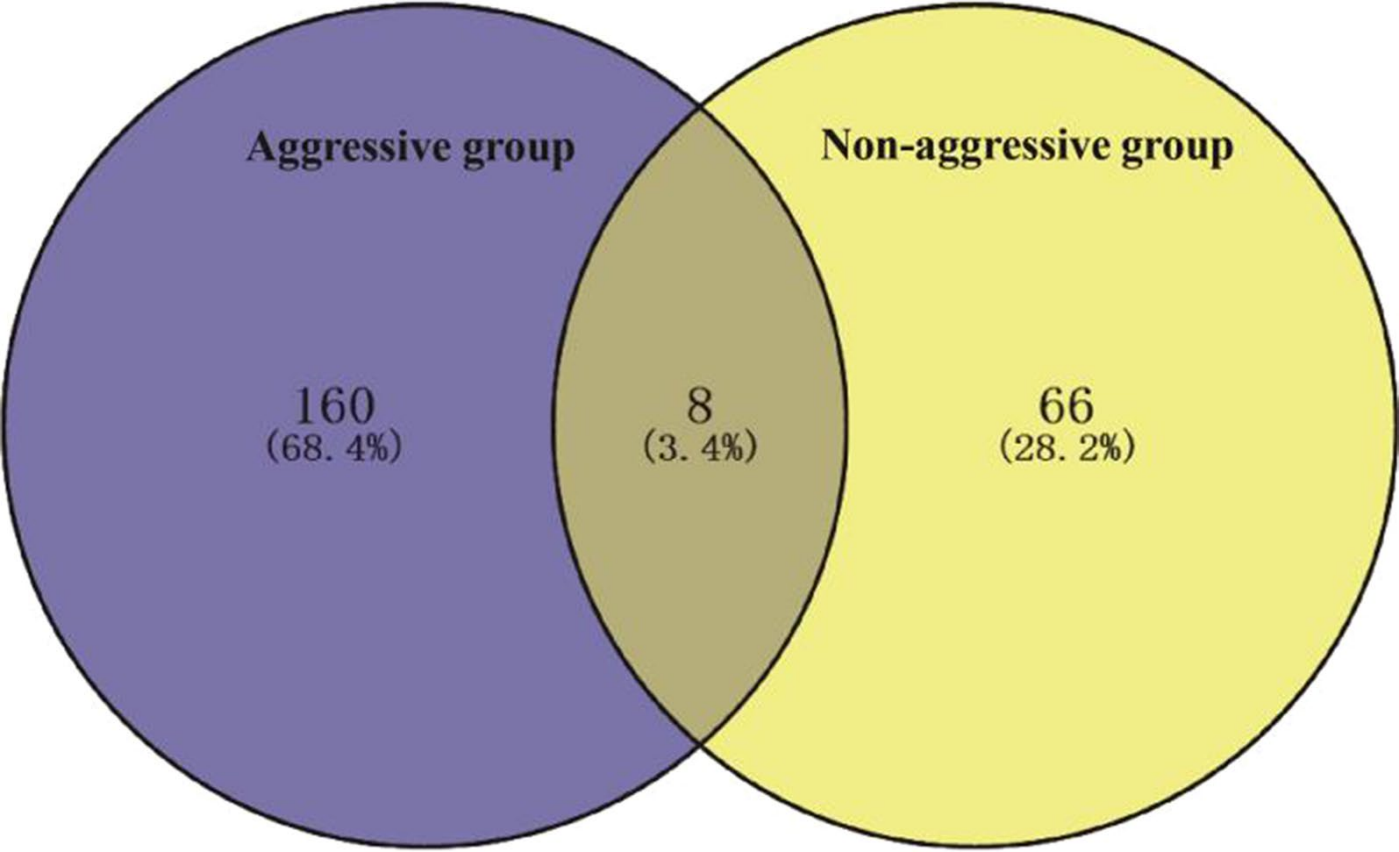
Venn diagram of somatic mutations in aggressive and non-aggressive groups

**Fig. 4:**
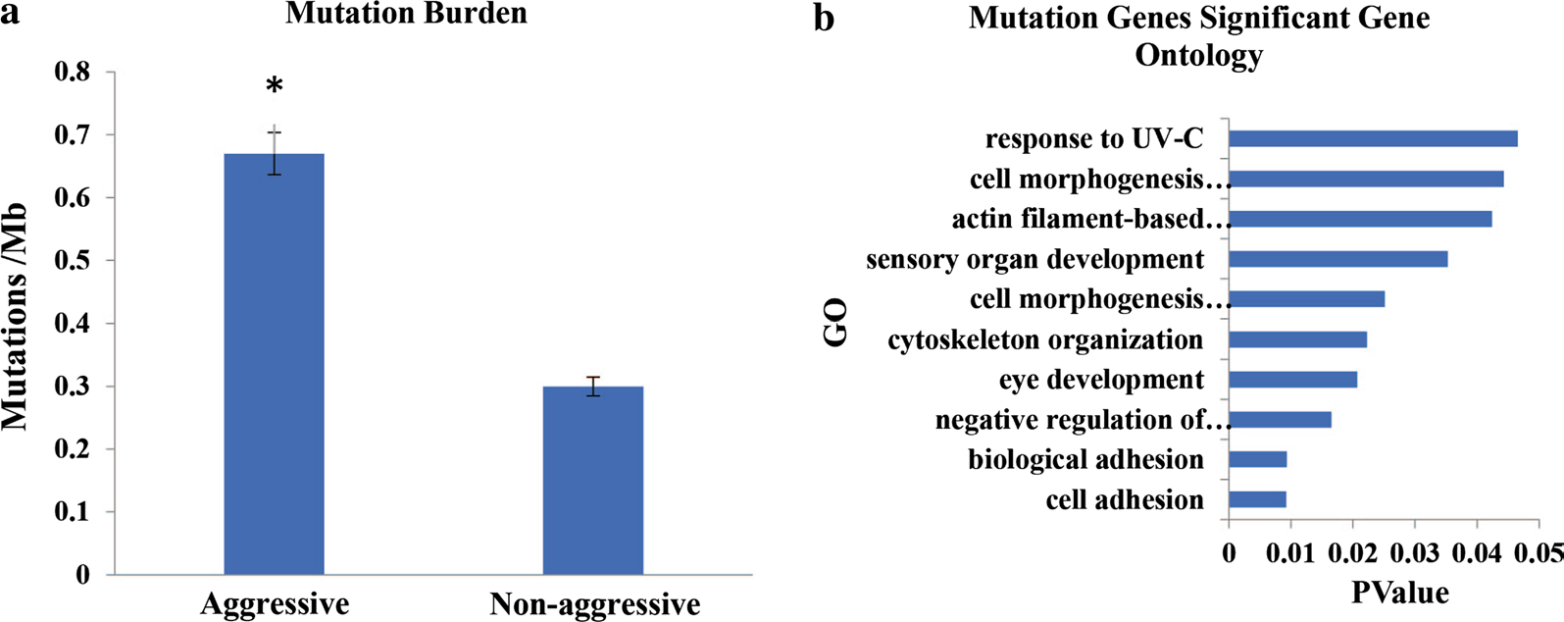
Mutational burden and biological functional analysis for both groups. **a** Difference in the TMB in aggressive and non-aggressive groups (P < 0.05). **b** GO analysis of mutations in aggressive group

We compared somatic mutations between aggressive and non-aggressive groups, and 8 mutated genes overlapped. The percent of different mutant gene sets in each group is depicted in Fig. 3. There were statistically significant differences in overall mutations between aggressive and non-aggressive groups (*P *= 0.004) (Fig. 4a).

### Key pathways and biological consequences related to PTMC aggressiveness

**Fig. 5:**
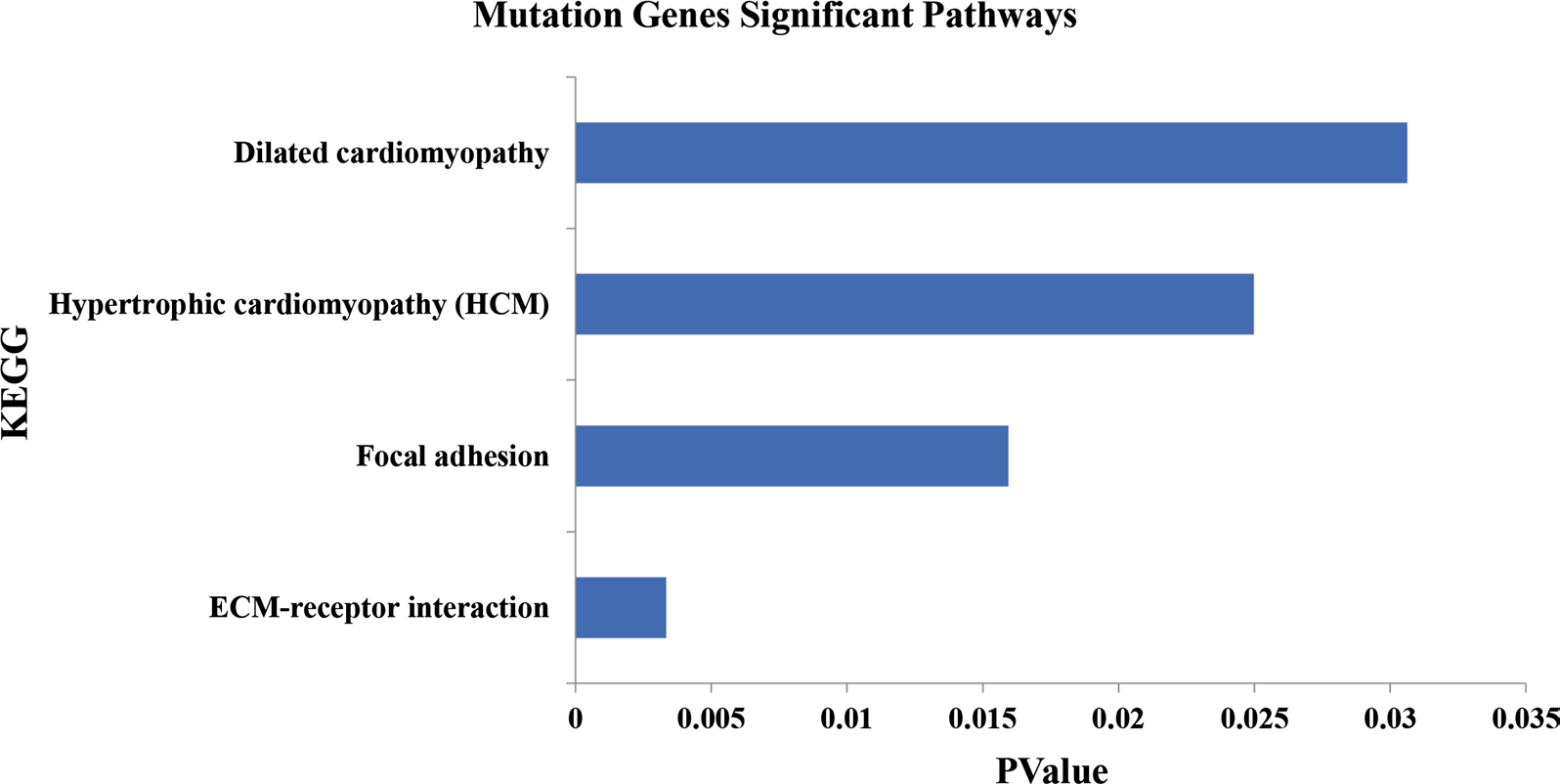
Pathways analysis of mutations in aggressive group

**Fig. 6:**
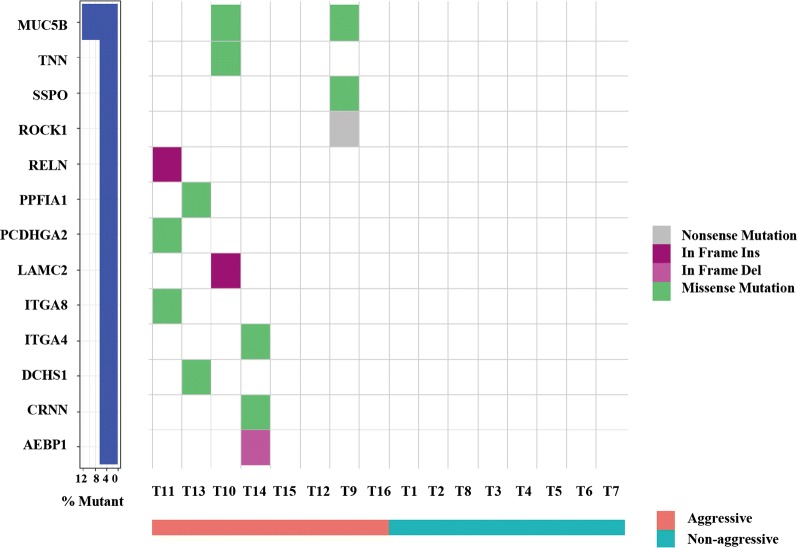
Mutations of cell adhesion genes in aggressive group. Mutations in 13 genes were identified in the aggressive group. Different mutations and subtypes are colored

GO and KEGG enrichment analysis used to analyze the pathways and biological functions for invasiveness and metastasis indicated heterogeneity within negative regulation of epithelial cell proliferation and alterations in multiple additional biological functions in the aggressive group (Figs. 4b, 5). Specifically, cell adhesion function was severely altered via mutations in 13 genes, and these data appear in Fig. 6.

### Validation cohort analysis of gene-panel targeted sequencing

**Fig. 7:**
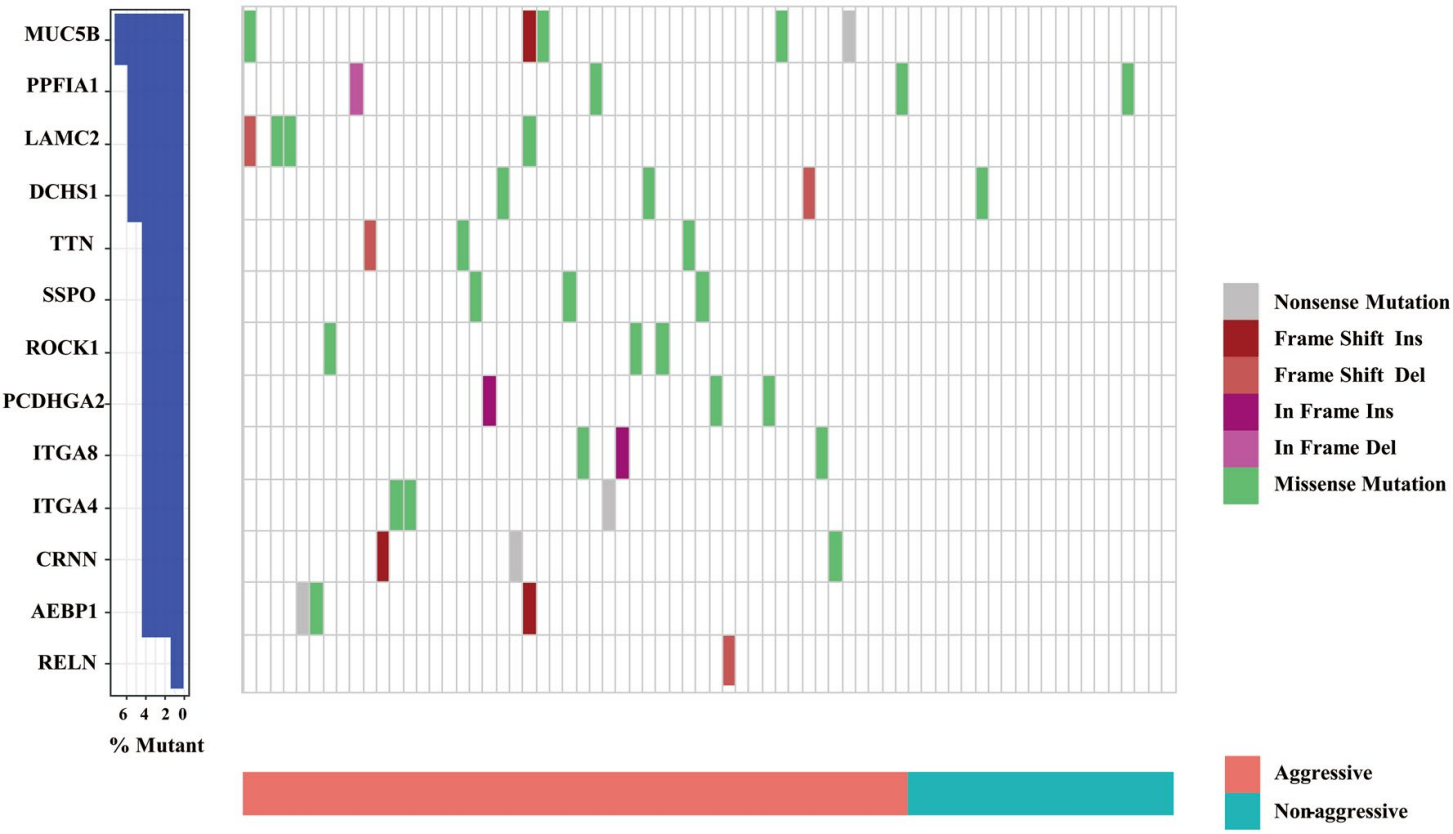
Frequency and types of mutations in 13 genes detected by targeted sequencing. Colors depict different types of mutations

Of 70 samples from 13-gene-panel targeted sequencing, 50 cases were considered aggressive PTMC due to extrathyroidal extensions or lymph node metastasis, and 20 cases were non-aggressive PTMC. In the aggressive group, there was at least one gene mutation in 37 cases (37/50), and in the non-aggressive group, only 2 cases had mutations (2/20). There was a significant difference between them (*P *= 0.000004) (Fig. 7).

## Discussion

Increased DTC is thought to be tied to PTMC, which is a focus of thyroid cancer research. We lack a method for predicting aggressive PTMC, and this hinders patient prognosis and treatment. Genetic analysis yielded insights related to altered gene mutations, signaling pathways, and biological functions of PTC, but such analyses are rare. Therefore, by understanding molecular biological characteristics, we may be able to better treat PTMC. Here we offer data regarding genomic profiles of key genes related to PTMC aggressiveness.

We used next-generation sequencing to find the prevalence of mutations in a cohort of PTMC and to identify unique patterns. WES was applied to 16 paired clinical specimens of PTMC, 8 of which were invasive and metastatic. The other 8 had no invasion and metastasis (non-aggressive). First, PTMCs in our WES cohort had an 0.49 overall mutation burden, suggesting it is “quiet” like PTC in TCGA [13]. Second, the mutation frequencies of *BRAF* and *NRAS* in PTMCs were 50% and 6.2%, respectively. Our data agree with previous work to describe driver mutations of PTC in TCGA [13, 15]. Third, of the 234 mutated genes identified in PTMC, multiple biological functions and pathways altered at high frequency were associated with them. For example, regulation of small GTPase-mediated signal transduction, regulation of Ras protein signal transduction, cell adhesion and ECM-receptor interaction, hypertrophic cardiomyopathy, and focal adhesion were included. Data show several common pathways for genetic aberrations for the most mutated genes in PTMC and PTC [13, 15–17]. Thus, we speculate that PTC evolved from PTMC because they share similar gene mutations and oncogenic signaling pathways.

Tumors arise, to a degree, from gene mutations, and the tumor mutation burden (TMB) is a measure of somatic mutations occurring in a tumor specimen. High TMB was associated with improved objective immune checkpoint inhibitor (ICPI) response and progression-free survival in lung and bladder cancer and for melanoma patients [18]. Therefore, TMB may be a genomic marker of prognosis and a predictor of treatment response [19]. To explore the relationship between TMB and aggressiveness, we analyzed differences in TMB between each group, and it was greater in the aggressive group, which is consistent with Robinson’s study regarding increased mutations for metastatic and primary tumors [20]. Therefore, the accumulation of mutations promotes PTMC aggressiveness and may be a focus of treatment.

We confirmed that *BRAF* was the most frequently mutated gene in PTMC and that it was associated with PTMC. Previously, *BRAF* mutations were associated with older age, tall-cell variants, extrathyroidal extensions, positive surgical margins, lymph-node metastasis, and stage IV disease [9, 10]. We found that BRAF^V600E^ was present in 8 tumor tissues (50%): 5 from the aggressive group and 3 from the non-aggressive group, indicating that *BRAF* mutations may not be correlated with invasion and metastasis. Because our study was limited to a small sample, validation of our preliminary data is required.

One particularly intriguing finding pertains to somatic mutations of cell-adhesion genes enriched in aggressive PTMCs. As cancer progresses and metastasizes, it often acquires new mutations. Core signaling pathways involved in nonsynonymous mutated genes (*MUC5B*, *TNN*, *SSPO*, *PPFIA1*, *PCDHGA2*, *ITGA8*, *ITGA4*, *DCHS1*, *CRNN*, *ROCK1*, *RELN*, *LAMC2*, and *AEBP1*) associated with cell and biological adhesion were identified in the aggressive group. Because tumor occurrence and development is dynamic and associated with adhesion, especially tumor cell migration [21], this may dictate the aggressiveness of PTMC. The targeted sequencing of the 13-gene panel in the subsequent expanded samples also verified the enrichment. Similar to ThyroSeq v3 [22], a DNA- and RNA-based next-generation sequencing assay that uses a genomic classifier (GC) to separate thyroid malignant from benign lesions, our 13-gene panel may have clinical utility for aggressive PTMC. Future research is needed with larger samples to confirm our preliminary data that cell adhesion related gene mutations can be used to predict PTMC aggressiveness and allow tailored treatment.

## Conclusion

PTC may have evolved from PTMC due to sharing similar gene mutations, and the accumulation of such mutations promoted PTMC aggressiveness. Somatic mutations of 13 cell-adhesion genes were identified to be uniquely associated with PTMC aggression. These candidate gene mutations may offer novel diagnostic and treatment options.
